# The Fungal Microbiome and Asthma

**DOI:** 10.3389/fcimb.2020.583418

**Published:** 2020-11-26

**Authors:** Erik van Tilburg Bernardes, Mackenzie W. Gutierrez, Marie-Claire Arrieta

**Affiliations:** ^1^ Department of Physiology and Pharmacology, Snyder Institute for Chronic Diseases, University of Calgary, Calgary, AB, Canada; ^2^ Department of Pediatrics, Alberta Children’s Hospital Research Institute, University of Calgary, Calgary, AB, Canada

**Keywords:** asthma, allergic responses, immune development, microbiome, mycobiome, environmental fungi

## Abstract

Asthma is a group of inflammatory conditions that compromises the airways of a continuously increasing number of people around the globe. Its complex etiology comprises both genetic and environmental aspects, with the intestinal and lung microbiomes emerging as newly implicated factors that can drive and aggravate asthma. Longitudinal infant cohort studies combined with mechanistic studies in animal models have identified microbial signatures causally associated with subsequent asthma risk. The recent inclusion of fungi in human microbiome surveys has revealed that microbiome signatures associated with asthma risk are not limited to bacteria, and that fungi are also implicated in asthma development in susceptible individuals. In this review, we examine the unique properties of human-associated and environmental fungi, which confer them the ability to influence immune development and allergic responses. The important contribution of fungi to asthma development and exacerbations prompts for their inclusion in current and future asthma studies in humans and animal models.

## Introduction

Asthma is one of the most common immune-mediated disorders affecting infants around the globe (Wong and Chow, 2008; Lai et al., 2009). Although a heterogeneous group of conditions, all asthma cases are characterized by chronic airway inflammation, bronchial hypersensitivity, and transient respiratory obstruction (Martinez and Vercelli, 2013). Asthma is routinely classified based on patient‘s immune status, with high serum levels of immunoglobulin E (IgE) or skin reactivity to common allergens in atopic asthmatics, and the absence of these in non-atopic patients (Pekkanen et al., 2012). Besides atopic/non-atopic classification, there are additional factors underlying asthma pathophysiology, resulting in distinct profiles of cellular infiltration in the airways, clinical symptoms, and treatments responses (Lotvall et al., 2011).

The immunology of asthma further highlights the complexity of this group of conditions. Asthma is classically considered an IgE-mediated, lymphocyte T helper 2 (Th2)-associated pathology, with an allergic inflammatory infiltrate characterized by eosinophils, mast cells, and CD4^+^ T cells producing interleukin-4 (IL-4), IL-5, and IL-13 in the airways (Martinez and Vercelli, 2013). However, increased immune profiling in asthmatics has revealed diverse disease immune patterns of Th1, Th2, Th9, Th17 T cell subsets, and mixed immune responses (Lambrecht and Hammad, 2015).

The factors that drive the development of asthma and the heterogeneity of its underlying immune responses are likely a combination of genetic and environmental influences. However, only environmental factors are likely able to explain the rapid and increasing societal burden imposed by asthma (van Tilburg Bernardes and Arrieta, 2017). Among these are factors directly and indirectly related to microbial exposures and perturbations during early life, such as respiratory infections (Stein et al., 1999; Busse et al., 2010; Olenec et al., 2010), antibiotic use (Marra et al., 2009; Russell et al., 2012; Patrick et al., 2020), birth by Caesarean section (Thavagnanam et al., 2008; Roduit et al., 2009; Darabi et al., 2019), reduced breastfeeding (Nagel et al., 2009; Kull et al., 2010), urban (vs. farm) upbringing (Wong and Chow, 2008; Lawson et al., 2011; Lawson et al., 2017), and pet exposures (Hugg et al., 2008; Fall et al., 2015). Human studies have linked these factors to distinct patterns of early-life microbial colonization that precede asthma and similar atopic disorders (Bisgaard et al., 2007; Arrieta et al., 2015; Fujimura et al., 2016; Arrieta et al., 2018), suggesting that the large community of microbes that colonize the intestinal and respiratory mucosae is an influential element in asthma pathogenesis.

While initial methods applied in microbiome studies mainly supported the survey of bacterial communities, advances in methodologies and extended curation of taxonomic reference databases to amplify, sequence, and classify the small subunit ribosomal RNA gene (18S) and internal transcribed spacer (ITS) marker have also allowed for the characterization of fungi within the microbiome (Huseyin et al., 2017; Hoggard et al., 2018). These have confirmed that, just like bacteria, fungi are also linked to asthma and atopy (Crameri et al., 2014; Fujimura et al., 2016; Arrieta et al., 2018; Goldman et al., 2018).

As ubiquitous members of terrestrial and aquatic ecosystems, fungi are part of the complex community of microorganisms that colonize mammalian epithelial and mucosal surfaces exposed to the environment (Nash et al., 2017; van Tilburg Bernardes et al., 2020). Current microbiome research has attributed fundamental roles to the bacterial microbiome in colonization resistance, nutrition, and providing neurological and immunoregulatory signals for normal host development [reviewed in (Dominguez-Bello et al., 2019) and (Cryan et al., 2019)]. However, the fungal microbiome, known as the mycobiome, has recently started to gain attention due to its important role in host health and disease (Huseyin et al., 2017).

**Figure 1:**
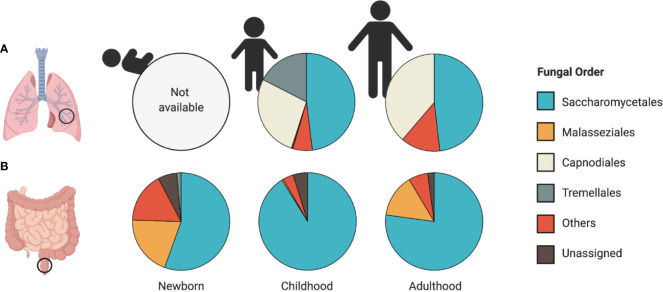
Patterns of airway and intestinal mycobiome development. Relative abundance plots of the most abundant fungal taxa at the Order level reported in **(A)** airway and **(B)** stool samples. **(A)** A lack of human studies assessing the mycobiome composition in the airways of newborns limits our understanding of early-life patterns of human airway colonization. Airway mycobiome studies in children (average age, 5.2 years old) and adults (average age, 35.7 years) revealed dominance by Saccharomycetales, along with a large proportion of environmental fungi from the Capnodiales order. **(B)** Early patterns of fungal colonization in neonatal stool reveal a high representation of Malasseziales and Saccharomycetales orders (average age 1 month). The stool mycobiome of children (average age 11 months) and adults (average age 27 years) is dominated by Saccharomycetales. Data presented from studies: **(A)** (van Woerden et al., 2013; Goldman et al., 2018) **(B)** (Fujimura et al., 2016; Nash et al., 2017; Ward et al., 2018).

Throughout infant development, and in parallel with the establishment of the bacterial microbiome, the mycobiome encounters ecological pressures and undergoes substantial compositional changes (**Figure 1**) (Fujimura et al., 2016; Laforest-Lapointe and Arrieta, 2017; Ward et al., 2018). Despite being outnumbered by orders of magnitude by the bacterial microbiome, the mycobiome elicits important immunomodulatory functions throughout early-life development (Kumamoto, 2016; Michalski et al., 2017; van Tilburg Bernardes et al., 2020). For example, our recent work showed that early-life fungal colonization distinctly altered innate and adaptive immune features, and impacted colitis onset and asthma development in gnotobiotic mice (van Tilburg Bernardes et al., 2020), supporting that fungal-derived microbial signals are important in host immune development.

Gut mycobiome studies in infant populations have found significant associations between mycobiome alterations and subsequent asthma and atopy susceptibility (Fujimura et al., 2016; Arrieta et al., 2018). Like bacteria, gut fungi also respond to environmental perturbations, such as antibiotic treatment (Samonis et al., 1993; Azevedo et al., 2015), which is a strong risk factor for asthma development in children (Marra et al., 2009; Martel et al., 2009; Murk et al., 2011; Arrieta et al., 2018). Further, several species of fungi are triggers of asthma and other atopic disorders (Denning et al., 2006; Agarwal and Gupta, 2011; Crameri et al., 2014), suggesting a role for fungi as part of the microbial exposures relevant in both asthma development and exacerbations. In the following sections we will review the current literature on gut, lung, and environmental fungi, their implications on asthma susceptibility and manifestations, the mechanisms by which fungi can mediate the development and persistence of this disease, as well as potential treatment options for asthma that target the mycobiome and mycobiome-induced immunity.

## The Intestinal Mycobiome

Sequence-based studies have allowed for the characterization of the intestinal mycobiome composition, defining its trajectory throughout life stages, and identifying associations with disease susceptibility. The first culture-independent study of the mycobiome in mammals revealed broad fungal diversity in the murine gut. Scupham et al. (2006), using oligonucleotide fingerprinting of ribosomal RNA (rRNA) genes, identified all major fungal phyla: *Ascomycota*, *Basidiomycota*, *Chytridiomycota*, and *Zygomycota* in the luminal content of laboratory mice (Scupham et al., 2006).

Further efforts to characterize these fungal communities led to a longitudinal study in 15 healthy children from Luxembourg that evaluated the intestinal mycobiome throughout the first year of life, using the 18S rRNA marker gene (Wampach et al., 2017). Stool from 1-day old babies revealed markable fungal diversity detected in meconium samples, suggesting that fungal colonization starts as early as bacterial colonization. From all microeukaryote operational taxonomic units (OTUs) identified in meconium samples, *Saccharomyces* spp. and *Exobasidiomycetes* spp. represented the most abundant and most frequently detected across samples, respectively (Wampach et al., 2017). Their work also demonstrated that, similar to bacteria, mode of birth influences fungal communities, with a higher abundance of *Saccharomyces* spp. and *Exobasidiomycetes* spp. in Caesarean-delivered infants, and higher *Dothideomycetes* spp. and *Pezizomycotina* in vaginally-delivered infants (Wampach et al., 2017). Although, in contrast with what has been described for bacterial succession patterns in the infant microbiome, fungi followed more aleatory temporal shifts in community composition, as evidenced by higher interindividual variation in fungal richness (number of OTUs), diversity (Shannon index), and evenness (Pielou’s evenness index) (Wampach et al., 2017). It is important to mention that most published microbiome results are based on comparisons of taxa relative abundance, which are notoriously biased, especially in samples with low microbial biomass. Wampach et al. (2017) demonstrated that samples with very low DNA yields, such as meconium, underestimated richness and altered relative abundance measurements in a 16S dataset. It is to be expected that similar deviations also occur in the mycobiome, especially considering their even lower biomass. Thus, additional methods to quantify total fungal DNA or taxa-specific DNA *via* quantitative PCR (qPCR) can provide necessary technical benchmarks to better interpret amplicon-based sequencing results.

In another study that included 17 healthy term Puerto Rican infants, the fecal mycobiome was dominated by only five fungal species: *Candida albicans*, *C. parapsilosis*, *C. tropicalis*, *Saccharomyces cerevisiae*, and *Cryptococcus pseudolongus*, with the first four species encompassing more than 10% relative abundance each (Ward et al., 2018). This study also showed high intraindividual diversity and no clear common trajectory toward a mature/differentiated community within 30 days of sampling (Ward et al., 2018), suggesting that there is a higher degree of stochasticity influencing the mycobiome composition during the initial colonization process of the human gut compared to bacteria.

A larger prospective study evaluated the mycobiome of 308 US infants using ITS2 sequencing (Fujimura et al., 2016). Participant age significantly impacted the fungal α- and β-diversity, with fungal α-diversity decreasing with age up to one-year. The neonatal mycobiome (~1 month of age) was dominated by *Malassezia* and *Saccharomyces*, and later shifted toward dominance by *Saccharomyces* and *Candida* at ~11 months of age (**Figure 1B**) (Fujimura et al., 2016). In contrast to the bacterial microbiome, which reaches full maturity after month 31 of life (Stewart et al., 2018), the fungal community composition is likely more stable after one year of age, as it is similar to the healthy mycobiome compositions reported in adults (Nash et al., 2017). However, this remains to be confirmed in longer longitudinal studies.

To characterize the composition of the adult intestinal mycobiome, Nash et al. (2017) sequenced the ITS2 and 18S genes in the first large mycobiome study in healthy adults, with a total of 317 participants. This study also highlighted the dominance of yeasts *Saccharomyces*, *Malassezia*, *Candida*, *Cyberlindnera*, *Pichia*, *Debaryomyces*, *Galactomyces*, and *Clavispora*, as well as the common presence of filamentous fungi, *Penicillium*, *Cladosporium*, *Aspergillus*, *Fusarium*, and *Alternaria* in the adult intestinal mycobiome (Nash et al., 2017). The high intra- and interindividual variation also reported in this study suggests that while fungi are common colonizers of the human gut, the specificity of the host-fungal species relationships may be less conserved or may involve less species compared to bacteria. This characteristic of the mycobiome introduces challenges to identify specific fungal taxa that may be associated with human diseases.

## The Airway Mycobiome

No longer considered sterile organs, the lungs harbor a diverse community of microorganisms, including fungi (Nguyen et al., 2015). There are very few studies looking into the fungal communities present in the lungs and airways of healthy individuals, and to our knowledge no studies have looked into these communities early in life to assess mycobiome establishment or associations with disease development **(** **Figure 1A** **)**. Work by Charlson et al. (2012) reported the presence of fungal communities in bronchoalveolar lavage (BAL) and oropharyngeal wash (OW) samples of six healthy individuals. Fungal communities in OW samples from these healthy volunteers were dominated by fungal species previously characterized in the oral cavity, including *Candida* and *Aspergillus* (Ghannoum et al., 2010). Fungi detected in BAL samples of these individuals revealed lower fungal amounts compared to OW, with several reads belonging to the genera *Davidiellaceae*, *Aspergillus*, *Penicillium*, and *Polyporales*, suggesting that fungal colonizers are present at lower concentration in the alveolar regions of the lungs compared to the upper airways (Charlson et al., 2012).

A study by Mac Aogain et al. (2018) further described the airway mycobiome composition in sputum samples of 238 bronchiectasis patients and 10 healthy controls from Asia and Europe. Fungal communities in the sputum of healthy controls were dominated by the *Ascomycota* phylum, with *Candida*, *Saccharomyces*, and *Meyerozyma* as the most commonly detected taxa (Mac Aogain et al., 2018). However, it should be noted that this study did not control for possible contamination of sputum samples with members of the oral mycobiome community, which frequently contains *Candida*, *Saccharomycetales*, *Cladosporium*, among others (Ghannoum et al., 2010). This important consideration is necessary for a reliable characterization of the respiratory mycobiome in infants and adults.

Several more studies have described fungal alterations in the context of chronic respiratory diseases, including asthma. Goldman et al. (2018) analyzed the mycobiome of BAL samples obtained from 15 severe asthmatic children and 11 non-asthmatic controls. Taxonomic analysis revealed 7 fungal genera differentially abundant between groups. Non-asthmatic children had increased abundance of *Davidiella*, *Cryptococcus*, and *Sterigmatomyces*, while children with severe asthma had increased abundance of *Rhodosporidium*, *Pneumocystis*, *Leucosporidium*, and *Rhodotorula* (Goldman et al., 2018). While informative and descriptive of microbial signatures in this asthmatic population, this study remains unable to determine if these specific airway mycobiome signatures are causally implicated in asthma development.

Mycobiome alterations have also been reported in asthmatic adults. A study by van Woerden et al. (2013) reported significant mycobiome compositional changes in induced sputum from 30 asthmatic adults and 13 healthy controls. A more recent study on endobronchial brush (EB) and BAL samples from 39 asthmatic adults and 19 healthy controls further associated mycobiota signatures with specific asthma phenotypes and clinical parameters (Sharma et al., 2019). This study found a decrease in fungal α-diversity (Shannon and Inverse Simpson indices), as well as an over-representation of *Trichoderma*, *Alternaria*, *Cladosporium*, *Penicillium*, and *Fusarium* in asthmatics, while health controls harboured increased abundances of *Blumeria*, *Mycosphaerella*, and different species from the *Fusarium* genera (Sharma et al., 2019). Interestingly, generalized linear regression models also found significant correlations between the abundance of exact sequence variants (ESV) of *Fusarium*, *Aspergillus*, *Cladosporium*, and *Alternaria* with Th2-high asthma (defined by blood eosinophil count >300/µL), *Cladosporium* and *Fusarium* with atopic-asthma (atopic status defined by allergic sensitization tests, skin prick test, or immunosorbent assay), and ESVs from Aspergillaceae, *Mycosphaerella*, and *Cladosporium* with non-atopic-asthma (Sharma et al., 2019). This important finding links distinctive airway mycobiome patterns with the pathophysiological mechanism of asthma. However, this and other microbiome studies in asthmatics (Chatterjee et al., 2008; van Woerden et al., 2013; Goldman et al., 2018; Sharma et al., 2019) are likely confounded by the inflammatory process of the disease itself, as well as its treatments, which can also differ according to asthma immune endotypes. Determining causality and directionality in these associations is important and requires prospective studies of children preceding and following asthma development, as well as longitudinal sampling of asthmatics during exacerbation and remission of asthma manifestations.

## Early-Life Fungal Exposures and Asthma Susceptibility

Observations from population-based and animal studies suggest that there is a critical window of opportunity, in which bacterial alterations during early life are important determinants of subsequent asthma susceptibility (Russell et al., 2012; Stiemsma and Turvey, 2017; Sokolowska et al., 2018). Unsurprisingly, the same applies to the mycobiome. Data from animal studies has demonstrated that common gut fungal colonizers can modulate the immune system and predispose the host to allergic airway inflammation. Antimicrobial-induced mycobiome alterations and intestinal expansion of *C. albicans* (Noverr et al., 2004; Noverr et al., 2005), *C. parapsilosis* (Kim et al., 2014), or *Wallemia mellicola* (Skalski et al., 2018) increased the severity of allergic airway inflammation in mice. Expansion of filamentous fungi (*A. amstelodami*, *Epicoccum nigrum*, and *W. sebi*) following antifungal treatment also exacerbated allergic airway responses in experimental mouse models (Wheeler et al., 2016; Li X. et al., 2018). Immune characterizations in these studies revealed that fungal-derived signals modulate airway inflammation by inducing alternative macrophage polarization to an M2 phenotype in the murine lungs (Kim et al., 2014), elevating RORγt^+^ and GATA3^+^ CD4^+^ T cells (Li X. et al., 2018), and increasing Th2 immunity in the airways (Noverr et al., 2004; Noverr et al., 2005; Wheeler et al., 2016; Li X. et al., 2018; Skalski et al., 2018). These studies suggest that alterations to gut fungal communities can increase host predisposition to airway allergic inflammation.

Prospective human cohort studies have investigated early life sensitization to fungal antigens on asthma development later in life. A longitudinal cohort study of 849 children by Stern et al. (2008) found that sensitization to *A. alternata* prior to six years of age was independently associated with chronic asthma by 22 years of age. Harley et al. (2009) reported that air levels of *Ascomycota* and *Basidiomycota* spores within the first 3 months of life are positively correlated with increased wheeze development, pointing to a possible role of airborne fungal spores in early-life immune sensitization events that lead to asthma.

Observations from prospective infant cohort studies that have investigated the gut mycobiome suggest similar implications for asthma susceptibility. Following observations from a Canadian cohort study which associated early-life microbial alterations with later susceptibility to asthma development (Arrieta et al., 2015), Arrieta et al. (2018) surveyed the bacterial and fungal microbiome in infants from a rural district in Ecuador. This study analyzed stool samples collected at 3 months of age, from 27 children with atopic/wheeze phenotype at 5 years of age and 70 healthy controls. Fecal samples from the infants that later developed atopic/wheeze revealed a higher proportion of total sequenced fungal reads, significantly increased fungal 18S recovered DNA, and overrepresentation of *Pichia kudriavzevii* at 3 months of age (Arrieta et al., 2018), suggesting a link between gut fungal overgrowth preceding asthma and revealing fungal alterations associated with increased child susceptibility to asthma development by school age.

Similarly, Fujimura et al. (2016) identified specific fungal genera correlated to an increased susceptibility to allergic asthma. Using a Dirichlet Multinomial Mixture model to separate participants into defined microbial community types, the study reported that a microbial profile depleted of *Malassezia* and enriched with Saccharomycetales, *Rhodotorula*, Pleosporales, and *Candida* had an increased relative risk of atopy diagnosis by two years of age, and parental reported doctor–diagnosed asthma at four years (Fujimura et al., 2016). This microbial composition was also associated with the pro-inflammatory metabolite 12,13-dihydroxy-9Z-octadecenoic acid (12,13-DiHOME). Sterile fecal water from this high-risk group and 12,13-DiHOME increased CD4^+^IL4^+^ T cells and decreased CD4^+^CD25^+^FOXP3^+^ T cells in *ex vivo* culture of human peripheral T cells (Fujimura et al., 2016). This strongly suggests that dysregulation in immune tolerance mechanisms that occur very early in life originate from what Fujimura et al. (2016) referred to as “interkingdom microbiota dysbiosis.”

**Figure 2:**
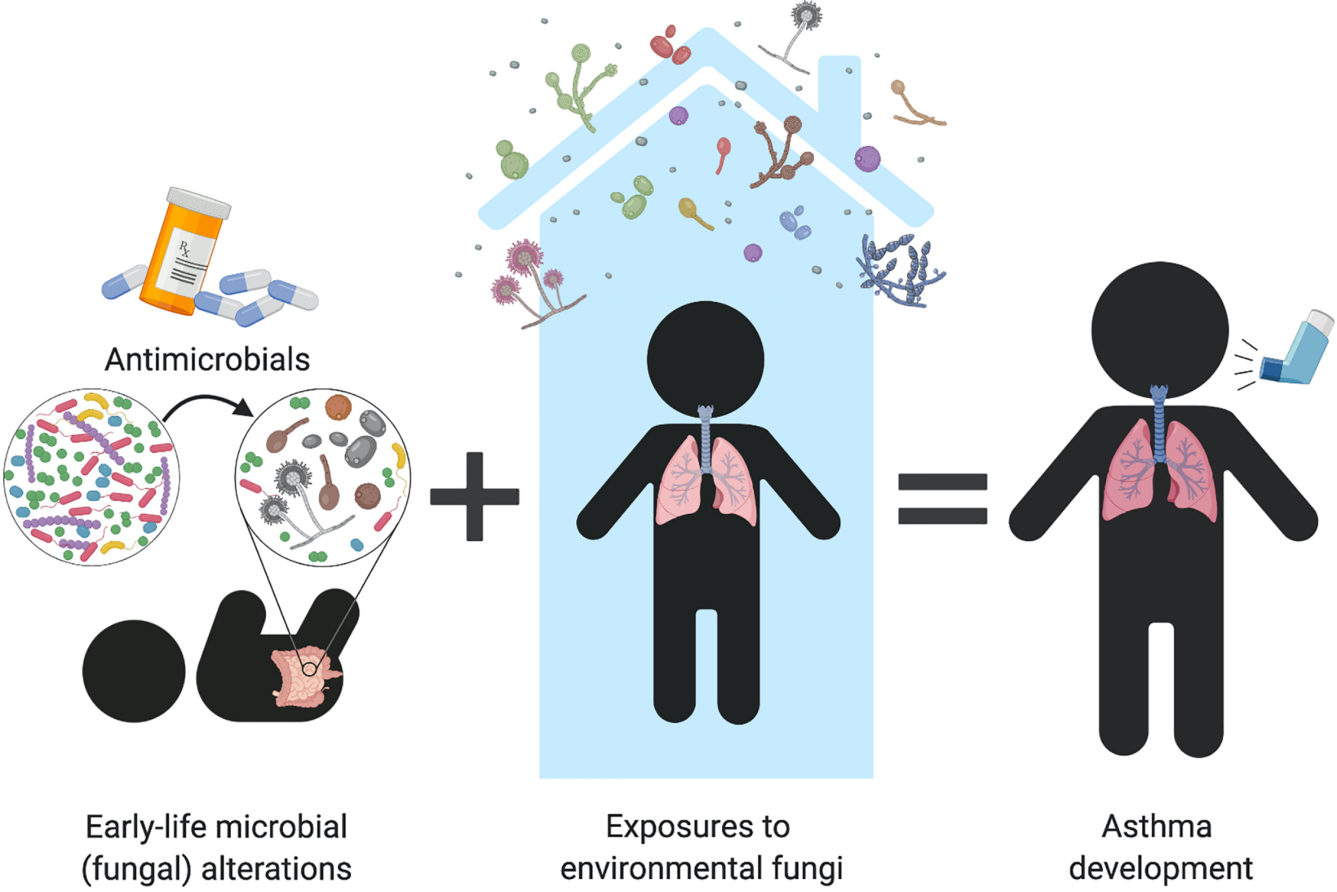
Can early-life fungal dysbiosis contribute to increased pediatric asthma risk? Early-life gut fungal alterations provide immunomodulatory signals that increase infant’s susceptibility to asthma. Infant antimicrobial use disrupts the intestinal microbiome and favors fungal overgrowth, which has been associated with allergic airway inflammation in both human and animal studies. Early-life mycobiome dysbiosis may contribute to dysregulated immune mechanisms that lead to immune sensitization to environmental fungi, resulting in fungi-triggered asthma in children and adults.

Both of these infant cohort studies (Fujimura et al., 2016; Arrieta et al., 2018) support that fungal alterations are an important part of the dysbiotic patterns predicting subsequent susceptibility to allergic diseases in children. Although majorly outnumbered by bacteria in the microbiome, this may imply that fungi provide important immune signals that, when imbalanced, can lead to immune dysregulation and pediatric asthma development. It remains to be determined how mycobiome alterations originate but it is possible that similar environmental factors known to induce bacterial perturbations are at play. For example, these imbalances may arise from antibiotic exposure, which are known to induce fungal dysbiosis and increase airway inflammation in animal studies (Noverr et al., 2004; Noverr et al., 2005; Kim et al., 2014; Skalski et al., 2018). Antibiotic use during early life has been repeatedly identified as a risk factor to atopy and asthma (Marra et al., 2009; Martel et al., 2009; Murk et al., 2011; Arrieta et al., 2015), suggesting that fungal overgrowth and/or dysbiosis may be part of the collateral microbiome damage caused by antibiotic use during infancy **(** **Figure 2** **).** This is further supported in humans by observations that antimicrobial induced fungal overgrowth was associated with an increased risk of allergic asthma development in infants (Arrieta et al., 2018). These early-life mycobiome alterations and fungal overgrowth following environmental perturbations might augment fungal immune recognition or allow for increased secretion of fungal-derived virulence factors. Recognition of these factors by immune cells may induce immunological imbalances that predispose infants to asthma development (further discussed in “Fungal-mediated immune mechanisms in asthma” section).

The increase in prospective and longitudinal infant gut microbiome studies, together with the inclusion of fungi in these surveys is consistently revealing gut bacterial and fungal alterations that may be involved in asthma pathogenesis. However, it is also likely that the lung mycobiome plays a role in altering lung immune responses. Because of the lack of animal or human studies examining the lung mycobiome and its relation to immune development, it remains unknown if succession patterns of lung fungal colonization during infancy, and alterations to these, may also contribute to asthma pathogenesis.

## Environmental Fungal Exposures and Asthma Development

Understanding the effect of diverse fungal exposures early in life is important because we are continuously in contact with environmental fungi which are capable of influencing human immune responses. Fungal spores are dispersed through indoor (Qian et al., 2012; Adams et al., 2013; Barberan et al., 2015) and outdoor air (Wyatt et al., 2013). Additionally, fungi have been detected in high levels on indoor surfaces, by both culture-dependent [25-130 CFU/mg of dust samples (Behbod et al., 2015)] and culture-independent [17-271 OTU detected in dust samples (Adams et al., 2013)] approaches. These environmental exposures likely shape the composition of the lung and gut mycobiome, as evidenced by a high representation of environmental fungi in the airways of adults (van Woerden et al., 2013), as well as the compositional changes in the intestinal mycobiome that parallels that of the surrounding environment (Dollive et al., 2013).

While several studies generalize fungal airway exposures as conducive to atopy and asthma development, others have shown that fungi may play a role in both reducing or increasing the risk of atopy and asthma in early life. For example, findings from Behbod et al. (2015) suggest that compartmental differences in exposure to fungi in early life may influence development of allergic asthma. In this study, increased overall yeast concentration in house dust from the homes of 408 infants of 2-3 months of age, was negatively associated with the development of wheeze and asthma by 13 years of age [hazard ratio (HR) = 0.88 and 0.86, respectively]. In contrast, indoor airborne *Alternaria* increased asthma risk (HR=1.48) (Behbod et al., 2015). The authors propose that this may result from the development of immune tolerance toward yeasts from gut exposures via hand-to-mouth behaviors during early life (Behbod et al., 2015), which may not occur to the same extent with airborne fungi. This interesting possibility remains to be tested experimentally.

In a systematic review of 61 studies conducted by Tischer et al. (2011), visible mould exposure was consistently associated with different reports of asthma, wheeze, and allergic rhinitis [odds ratio (OR) = 1.49, 1.68, and 1.39, respectively]. However, when fungal exposure was further stratified to evaluate specific fungal components, (1,3)-β-D-glucan and extracellular polysaccharides, ubiquitously secreted by most fungal species, were inversely associated with wheeze and asthma in children (Tischer et al., 2011), highlighting that fungal exposure should not be aggregated as a single study variable. Altogether, these findings prompt for more robust methods to investigate the epidemiology and mechanisms by which environmental fungal exposures in early life may influence asthma development or protection.

In addition to asthma development, fungal environmental exposures have also been consistently linked to specific asthma phenotypes. A study by Dannemiller et al. (2016a) found asthma severity and atopic status linked to higher fungal detection in house dust. This cohort of 587 asthmatic children in Connecticut and Massachusetts, revealed a positive association between asthma severity and increased summed concentration of fungal species, including *A. alternata*, *C. albicans*, *C. cladosporioides*, *P. brevicompactum*, *M. sympodialis*, and *R. mucilaginosa* (Dannemiller et al., 2016a; Dannemiller et al., 2016b). Notably, while fungal community composition was not different among the houses of children with mild or severe non-atopic asthma, fungal communities from houses of children with mild atopic asthma had higher abundance of fungi from genus *Vellutella* (Dannemiller et al., 2016a). These observations provide support for the role of environmental fungi as environmental cues capable of modulating the tone and immune phenotype of asthma in children (**Figure 2**). While experimental evidence in animal models is still needed to confirm causality in these observations, the environmental nature of these exposures renders them less susceptible to biological confounding effects, and strongly suggest that these may be involved in the initial allergic sensitizations that lead to asthma development.

## Fungi in Asthma Exacerbations

Not only does fungal exposure during early life play a role in asthma development, but fungal exposures are also well known to induce or exacerbate episodes in asthmatic patients. A large study that included 831 US homes found *A. alternata* in house dust to be correlated with active asthma symptoms (Salo et al., 2006). Sharpe et al. (2015) also identified widespread associations between indoor presence of *Cladosporium*, *Alternaria*, *Aspergillus*, and *Penicillium* and increased asthma exacerbation in adults and children through random-effect estimates in a meta-analysis of 7 studies, suggesting that fungal exposures are conducive to asthma exacerbations, and have implications for disease severity and management.

Sensitization due to previous exposure to specific fungal species is likely to play a role in subsequent exacerbations. In a study on adults hospitalized for asthma, Niedoszytko et al. (2007) found associations between skin prick test sensitization to *Helminthosporium* and *A. pullulans* with increased asthma exacerbations and severity, respectively. Another study in adult asthmatics that harbored *A. fumigatus* in sputum reported an association between *A. fumigatus*-IgE sensitization and asthma severity, neutrophilia, and reduced lung function (Fairs et al., 2010).

A number of studies have also investigated the relationship between exposure and sensitization to fungi and asthma exacerbation during childhood. A study of 280 children from 37 inner-city schools in Boston, U.S.A. found that exposure to *Alternaria* in classrooms was associated with increased duration of asthma symptoms in children already sensitized to *Alternaria*, compared to sensitized children with a lower classroom exposure level over a 2-week period (Baxi et al., 2019). Welsh et al. (2016) cultured the sputum from children with either acute exacerbation or stable asthma, and found increased concentrations of *A. fumigatus* in the sputum from children experiencing exacerbation, suggesting a role of this fungal species in asthma manifestations. Similarly, the Melbourne Air Pollen Children and Adolescent study found associations between asthma hospitalizations, outdoor total fungal spores, and levels of *Alternaria*, *Leptophaeria*, *Coprinus*, and *Drechslera*, in 644 asthmatics between the ages of 2 and 17 (Tham et al., 2017).

From these studies, it is clear that fungi play an important role in the exacerbation of asthma in children and adults, and that these often emerge from immune sensitization during infancy or childhood. Nonetheless, not all exposures to fungi result in asthma-inducing sensitizations, indicating that, just like with bacteria, many may indeed be protective, and that the underlying mechanisms of host-fungal immune crosstalk stem from the immunogenicity of specific fungal species and/or host immune susceptibilities. While it is evident that not all fungal exposures are detrimental to immune development, certain species, such as those belonging to the genera *Alternaria* and *Aspergillus*, are consistently associated with sensitization in asthmatics and asthma severity. As such, further research on the microbial and host immune mechanisms relevant to asthma pathogenesis should include fungi, both protective and harmful species, from the human microbiome and the environment.

## Fungi as Unique Allergens

**Figure 3:**
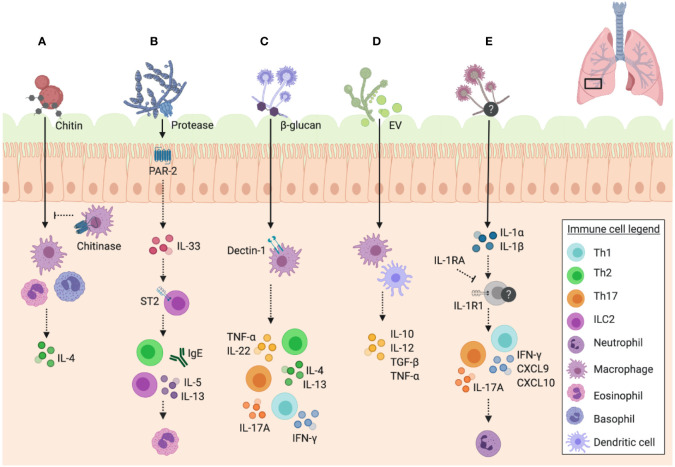
Unique features of fungal immune responses in the airway. Schematic of the unique features of fungi and their interactions with immune mechanisms in the murine airway. **(A)** Chitin in fungal cell walls triggers alternative activation of macrophages (M2 phenotype) and IL-4 expressing eosinophil and basophil responses, which can be inhibited by mammalian chitinases. **(B)** Fungal proteases signal through PAR-2 in the airway epithelium resulting in the release of IL-33, which consequently signals through ST2 to trigger IgE and ILC2 release of IL-5 and IL-13, promoting eosinophil recruitment to the airway. **(C)** β-glucan recognition through dectin-1 on macrophages results in the release of TNF-α, as well as a mixed Th1/Th2/Th17 response with the release of IL-4 and IL-13 by Th2 cells, IFN-γ by Th1 cells, IL-17A by Th17 cells, and IL-22 detected in unfractionated lung cells. **(D)** Extracellular vesicles (EV) trigger the release of cytokines IL-10, IL-12, TGF-β, and TNF-α by macrophages and dendritic cells. **(E)** *A. fumigatus*-induced allergic airway inflammation in mice induces IL-1R1 signalling and Th1 (IFN-γ, CXCL9, and CXCL10) and Th17 responses, with IL-17A promoting neutrophil infiltration. IL-1RA inhibits these pathways downstream of IL-1R1. Data presented from studies: **(A)** Reese et al., 2007; Van Dyken et al., 2011; **(B)** Bartemes et al., 2012; Snelgrove et al., 2014; Castanhinha et al., 2015; Hiraishi et al., 2018 **(C)** Gersuk et al., 2006; Lilly et al., 2012; **(D)** Vargas et al., 2015; and **(E)** Godwin et al., 2019.

Why are certain fungi and their structures so frequently associated with asthma and other allergic diseases? While this remains unknown, fungi have unique properties that may provide them with the ability to increase asthma susceptibility and induce exacerbations in the host (**Figure 3**). Whether this comes from earlier sensitization followed by subsequent hypersensitivity, or through direct immunomodulatory properties of fungi remains under debate. A study by Zhang et al. (2017) suggests that the ability of certain fungi to exacerbate asthma symptoms is independent of their capacity to act as initial sensitization allergens, and is reliant on the immunomodulatory abilities of certain fungal components. Specifically, the authors found that children in the Cincinnati Childhood Allergy and Air Pollution Study showed an association between fungal exposure and asthma prevalence in the absence of fungal sensitization (Zhang et al., 2017). These findings were further validated in mice, in which direct exposure to *A. versicolor*, without previous sensitization, resulted in enhanced airway inflammation in a house dust mite (HDM) model (Zhang et al., 2017).

Chitin, the second most common polysaccharide in nature (after cellulose), plays an important role in the development of asthmatic disease following airway exposure to fungi. Chitin is a major component of fungal cell walls, helminths, insects, and crustaceans, but it is not present in mammals (Lee et al., 2008). While mammals express chitinases to enzymatically break down chitin, the biological role of these enzymes in host biology remains incompletely understood. Mammalian chitinases are expressed at inflammation sites, and thus are hypothesized to play roles in host anti-microbial and anti-parasite responses (Lee et al., 2008). Interestingly, both chitin and chitinases are implicated in the pathophysiology of asthma, including fungal asthma (Lee et al., 2008; Shuhui et al., 2009).

A study by Van Dyken et al. (2011) found that while chitin induces an eosinophilic response, mice constitutively expressing a mammalian chitinase are able to limit eosinophilic infiltration following challenge with a fungal preparation derived from house dust (**Figure 3A**). Another study investigated the role of acidic mammalian chitinase (CHIA) in atopic asthma, through detection of polymorphisms of this gene in asthmatic patients in Northern India. The authors identified three specific promoter polymorphisms associated with atopic asthma, total serum IgE, or both, confirming the role of CHIA as an asthma susceptibility gene (Chatterjee et al., 2008). Additionally, Ober et al. (2008) identified *CHI3L1*, the gene encoding a chitinase-like protein (YKL-40) that had been previously associated with asthma severity, as another susceptibility gene for asthma. They specifically identified a single nucleotide polymorphism in the *CHI3L1* promoter region that could be used to predict asthma and serum YKL-40 levels (Ober et al., 2008). Similarly, a study by Wu et al. (2010) was able to identify an association between fungal exposure and specific chitinase single nucleotide polymorphisms in the context of asthma exacerbation as measured by emergency room visits. The relationship between the role of chitinases in recognizing fungi and the genetic susceptibilities of specific chitinase genes in asthma development hint at the capacity of fungi to elicit allergic airway responses, as well as the crucial role of chitinases in the resolution of this inflammation.

It has also been proposed that asthma occurs as a result of the Th2 reaction to fungi as a means to contain what the immune system recognizes as a fungal infection, and that fungal proteases are crucial to this response. The study by Porter et al. (2009) investigated the role of active proteases in the house dust of asthmatic children, in eliciting an airway response in mice. The study established that it was specifically fungal proteases, particularly from *A. niger*, which were found to be active in the house dust as opposed to proteases from other sources, such as mites (Porter et al., 2009). They also established that these proteases are required in combination with *A. niger* conidia to elicit a complete asthmatic disease phenotype, and that protease mutants are able to thrive based on a deficient ability to elicit an airway immune response characteristic of asthma (Porter et al., 2009). This emphasizes the role of unique fungal proteases in asthmatic disease.

A study by Snelgrove et al. (2014) was able to elucidate the underlying immunomodulatory mechanism of *A. alternata* serine proteases using a mouse model. They found that protease activity was absent in other common aeroallergens, and that the serine protease of *A. alternata* possesses signalling capabilities through protease activated receptor-2 (PAR-2) and adenosine triphosphate that results in the rapid airway release of IL-33 (**Figure 3B**). This was responsible for subsequent Th2 airway inflammation and other hallmarks of asthma exacerbation (Snelgrove et al., 2014). Interestingly, it has been found that PAR-2 is upregulated in the airway epithelium of asthmatics relative to healthy controls (Knight et al., 2001), an important piece of evidence for the role of fungal proteases in asthma exacerbation and the possibility of underlying genetic susceptibilities to fungal asthma. Furthermore, alkaline protease 1 (Alp1) of *A. fumigatus* was shown to directly disrupt interactions between airway smooth muscle cells and the extracellular matrix, resulting in airway hyper responsiveness in mice challenged with *A. fumigatus* extract with active Alp1 (Balenga et al., 2015). To investigate these findings in humans, lung samples were stained for Alp1, which was detected in the airway smooth muscle layer of non-atopic asthmatic patients (Balenga et al., 2015). A negative association was found between Alp1 levels and lung function (Balenga et al., 2015), hinting at the role of fungal proteases in airway disease. The underlying mechanisms of fungal protease ability to elicit airway diseases are slowly being uncovered, and are just one important aspect of fungi that makes them distinct to other aeroallergens.

Fungal immunogenicity in the airways may also depend on the life cycle stage of sporulating fungi at the point of exposure. Environmental fungi are often introduced into the airway as resting spores, and may not elicit an immune response until present in metabolically active germinating form. A study by Gersuk et al. (2006) found that alveolar macrophages are capable of distinguishing between resting and active forms of *A. fumigatus*, and that this process relies on recognition of cell wall β-glucans by the innate host receptor dectin-1 on macrophages (**Figure 3C**), resulting in tumor necrosis factor-α (TNF-α) production in order to limit inflammatory responses to active cells. The authors propose that this may be a protective immunological mechanism in order to prevent tissue damage in the absence of a true infectious threat (Gersuk et al., 2006). The ability to control allergic immune responses depending on their metabolic state is another distinct feature of fungi, which likely confers an advantage to evade immune surveillance.

Like all existing cell types, fungi also have the capacity to secrete extracellular vesicles (EV), which may contribute to their immunomodulatory capacity for asthma development. EV carry enormous amounts of antigenic molecules, are known to modulate immune responses, and due to their elevated presence in experimental asthma models, are proposed to be involved in asthma pathogenesis (Nazimek et al., 2016). Joffe et al. (2016) refer to fungal EV as “virulence bags” based on their ‘cargo’, and highlight their potential use in vaccine development. Furthermore, Vargas et al. (2015) investigated the immunomodulatory activity of EV from *C. albicans*, a host-associated species with known links to asthma and allergic airway responses (Noverr et al., 2004; Noverr et al., 2005; Fujimura et al., 2016; van Tilburg Bernardes et al., 2020). The authors found that *C. albicans* EV are internalized by both macrophages and dendritic cells and stimulate the release of nitric oxide by macrophages and cytokines such as IL-10, IL-12, transforming growth factor-β (TGF-β), and TNF-α by both cell types (**Figure 3D**) (Vargas et al., 2015). While different studies show the implications of environmental bacteria-derived and host immune cell-derived EV in asthma pathogenesis (Nazimek et al., 2016), the immunomodulatory role of fungal-derived EV has yet to be investigated in the context of asthma.

**Figure 4:**
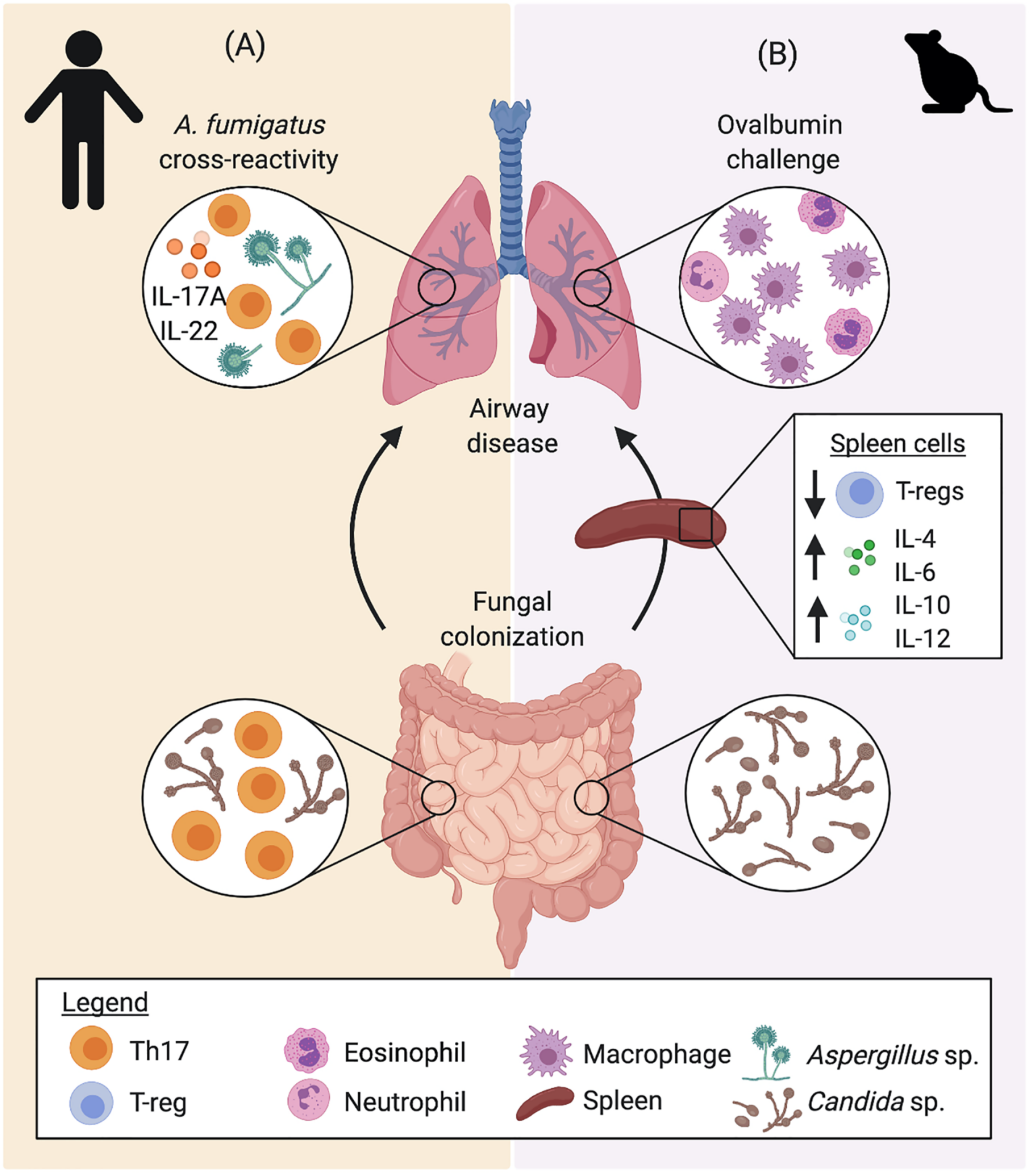
The gut-lung crosstalk in airway inflammation. Gut, systemic, and lung immune responses to *Candida* sp. colonization have been described in **(A)** humans and **(B)** mice, indicating that intestinal fungi impact immune development and that airway immune responses to other fungi and allergens (ovalbumin), respectively. **(A)** Protective CD4^+^ memory T cells generated from colonization by *C. albicans* secrete elevated levels of IL-17A and IL-22. Upon exposure to *A. fumigatus*, homology between fungal antigens directs the selective expansion of *A. fumigatus*-reactive cells, driving Th17 airway inflammation. **(B)** In mice, intestinal fungi colonization alters early life immune development, inducing increased systemic levels of IL-4, IL-6, IL-10, and IL-12, while reducing the proportion of Tregs. Colonization with fungi also increased airway inflammation in an ovalbumin challenge model, altering the inflammatory phenotype with increased macrophagic infiltration in BAL fluid. Data presented from studies: **(A)** Bacher et al., 2019; and **(B)** van Tilburg Bernardes et al., 2020.

A number of studies have looked into cross-reactivity between fungal species and the implication this has on airway disease. An early study by Hemmann et al. (1997) identified cross reactivity between *A. fumigatus* and *C. boidinii*, due to antigenic similarity, as well as *Candida* antigen’s capacity to bind to IgE from *A. fumigatus* sensitized individuals. Furthermore, Bacher et al. (2019) established an important link between fungal colonization in the gut with susceptibility to fungal mediated airway inflammation. The authors found that *C. albicans* are the main drivers of the development of anti-fungal Th17 cells and provide protective anti-fungal Th17 responses to *C. albicans* in the gastrointestinal tract (Bacher et al., 2019). While *C. albicans-*induced Th17 response has been shown to protect from intestinal and systemic infection (Shao et al., 2019), a subset of these *C. albicans*-reactive Th17 cells are able to cross-react with *A. fumigatus* due to shared homology between certain antigenic peptides, resulting in a pathogenic Th17 response in the airway (**Figure 4A**) (Bacher et al., 2019). The study demonstrated expansion of cross-reactive Th17 cells in the lungs of patients with acute allergic bronchopulmonary aspergillosis, which contributes to exacerbation of airway hyperreactivity (Bacher et al., 2019). Interestingly, cross reactivity has also been proposed between fungal and human antigens. Denning et al. (2006) highlight the homology between a number of fungal and human proteins that result in a cross reactive response between fungi and self-antigens of the host, resulting in an auto-reactive response, which may further exacerbate asthma. Cross reactivity between different fungal species may be an important feature explaining asthma exacerbations to a wide variety of fungi. Importantly, this feature may also imply that the immunological responses to common fungal intestinal colonizers early in life play a fundamental role in airway hyperreactivity. This is further evidenced by data from our group, demonstrating that intestinal fungal colonization induces early-life immune changes, characterized by altered systemic levels of inflammatory cytokines IL-4, IL-6, IL-10, and IL-12, and regulatory T cells (Tregs), which exacerbates airway responsiveness with elevated macrophagic infiltration in an ovalbumin (OVA) challenge mouse model (**Figure 4B**) (van Tilburg Bernardes et al., 2020). Cross reactivity of immunological responses to fungal antigens with human proteins or environmental molecules also establishes fungal colonization as an important factor in the development and manifestation of asthma.

## Fungal-Mediated Immune Mechanisms in Asthma

The underlying immunological mechanisms that render fungi elicitive of asthmatic disease are highly complex, and there is likely an interplay among the many features of fungi that may help explain the diverse immunopathology of this disease. Allergic asthma is characterized by a Th2 response (Martinez and Vercelli, 2013), consistent with that seen in fungal asthma models. A study by Hoselton et al. (2010) found elevated serum IgE and pulmonary IL-4 levels along with a dramatic increase in leukocyte infiltration following *A. fumigatus* airway challenge of previously sensitized mice. Mannose-binding lectin (MBL) has also been shown to be crucial for early Th2 responses in an *A. fumigatus* mouse model of airway inflammation, such that mice deficient in MBL-A displayed a dampened Th2 cytokine response as well as pro-allergic chemokine expression (Hogaboam et al., 2004). Similarly, chitin found in fungal cell walls is associated with the induction of type 2 inflammation. Reese et al. (2007) found chitin exposure in the airways to be associated with recruitment of IL-4-expressing innate immune cells, eosinophils, and basophils, along with an M2 macrophage phenotype in mice relative to a saline-challenged control (**Figure 3A**). An increased chitin to β-glucan expression ratio in *A. fumigatus* strains resulted in greater eosinophil recruitment in a mouse model of airway inflammation, dependent on γδ-T cells, indicating the role of this T cell subset in the type 2 response to chitin (Amarsaikhan et al., 2017). Using *Ccr1* ^-/-^ mice, Blease et al. (2000) showed that C-C Motif Chemokine Receptor 1 (CCR1) signalling is necessary for Th2 responses and airway remodeling in a *A. fumigatus* model of airway inflammation, another mechanism by which fungi are capable of eliciting such airway response.

While Th2 responses are a hallmark of allergic asthma, they are just one component of the immune response involved in fungi driven asthmatic disease. IL-33 is an innate cytokine commonly found in the airways to be associated with asthma, and has been shown to be involved in group 2 innate lymphoid cell (ILC2) development (Bartemes et al., 2012) and airway remodeling with steroid resistance (Saglani et al., 2013). Mice exposed to *A. alternata* showed an elevation of IL-5 and IL-13 derived from ILC2 along with eosinophilia (**Figure 3B**), which was diminished in the absence of ST2 (IL-33 receptor) signalling, further implicating innate immune mechanisms in fungal asthma (Bartemes et al., 2012). Children with severe asthma with fungal sensitization (SAFS) have greater IgE titers and an association with elevated IL-33 (Castanhinha et al., 2015). When investigated in a neonatal mouse model, *A. alternata* exposure resulted in greater airway IL-33, IL-13-producing ILC, and Th2 cells along with elevated serum IgE independent of corticosteroid treatment, compared to HDM exposure (Castanhinha et al., 2015). These effects were diminished in mice lacking a functional IL-33 receptor (*ST2^-/-^*), suggesting IL-33 as a primary mediator of innate responses in SAFS and an important factor in the resistance to corticosteroid therapy experienced by SAFS patients (Castanhinha et al., 2015). These findings provide evidence for the involvement of innate immunity in fungi-associated asthma development and severity, alongside the well-established role of adaptive immunity.

As previously mentioned, proteases are a unique feature of fungi that contribute to the pathology of fungi-associated asthma, and these enzymes in turn contribute to the specific immunological mechanisms induced in response to fungal airway exposure. Inhaled *Aspergillus-*derived fungal associated proteases (FAP) induced airway eosinophilia in naive mice through protease activated receptor-2 (PAR-2), indicating that this is directly associated with protease activity rather than sensitization (Hiraishi et al., 2018). IL-33 induced by PAR-2 signaling played a crucial role in this response through activation of ILC2 (independent of adaptive immunity) to drive the eosinophilic inflammation seen (**Figure 3B**) (Hiraishi et al., 2018). This research highlights key innate immune mechanisms by which fungal airway exposure without sensitization can be conducive of steroid resistant asthma.

Th1 responses may also play an important role in fungi-associated asthma, despite the classic predominance of type 2 responses associated with this disease. In an *A. fumigatus* allergic airway model, along with elevated Th2 responses, mice displayed an increase in interferon-γ (IFN-γ), a well characterized Th1 cytokine, upon airway challenge (Hoselton et al., 2010). Godwin et al. (2019) found that the BAL fluid of asthmatic patients with fungal sensitization displayed an elevation in IL-1α and IL-1β, and with an *A. fumigatus* model of airway inflammation, they established that IL-1R1 signaling is associated with Th1 and Th17 responses, which contributes to the severity of fungal asthma (**Figure 3E**). Using *Il1r1^-/-^* mice, the authors showed that IL-1 plays an important role in neutrophil and eosinophil recruitment to the airway and thus underlies the severity of the inflammatory response (Godwin et al., 2019). They also reported that interleukin-1 receptor antagonist (IL-1RA) treatment reduced Th1 associated IFN-γ, C-X-C Motif Chemokine Ligand-9 (CXCL9), and CXCL10 along with Th17 associated IL-17A, which were directly associated with neutrophil recruitment (Godwin et al., 2019).

Beyond Th1 and Th2 responses, Th17 responses have been shown to play a role in fungi-associated asthmatic disease in response to fungal airway exposure. As previously mentioned, Bacher et al. (2019) displayed a model in which *C. albicans*-reactive T cells drive Th17 responses in the lung against *A. fumigatus* (**Figure 4A**). Recognition of *A. fumigatus via* the dectin-1 receptor in the airway has also been shown to be associated with IL-17A and IL-22 production by CD4^+^ T cells and unfractionated lung cells, respectively, which results in worsened allergic airway response in a murine model (Lilly et al., 2012). However, dectin-1 signaling was also shown to be associated with CD4^+^ T cell production of IL-4, IL-13, and IFN-γ to a lesser extent (**Figure 3C**), suggesting an interplay between Th2 and Th17 responses in fungi induced asthma (Lilly et al., 2012). Similarly, Zhang et al. (2017) reported that severe steroid resistant fungal asthma in mice displays a mixed Th2 and Th17 response in a HDM/*A. versicolor* combination model, which is not seen with HDM alone. Co-exposure was associated with both neutrophilia and eosinophilia, highlighting the basis for the steroid resistant phenotype of this model (Zhang et al., 2017).

IL-17A is associated with neutrophil recruitment (Newcomb and Peebles, 2013), which is a hallmark of severe asthma (Fahy, 2009), and may play a direct role in the enhanced severity experienced by patients with fungi associated asthma. This phenomenon was shown in an *A. fumigatus* mouse model of fungal allergic airway disease, whereby decreased recruitment of neutrophils ameliorated airway hyper responsiveness (Park et al., 2006). In order to understand the underlying mechanisms by which fungal exposure results in severe asthmatic disease, it is crucial that other arms of the immune system beyond a classic type 2 allergic response be taken into consideration.

While environmental exposures may explain differences in allergic airways responses, a genetic component has been implicated in fungal-associated immune responses conducive of asthma in children. Knutsen et al. (2010) investigated T cell responses in children with *Alternaria-*sensitive mild and moderate-to-severe asthma and found that the moderate-to-severe group showed increased expression of IL-5 and IL-13 by T cells stimulated with *Alternaria ex-vivo*. Notably, the moderate-to-severe group had a significantly greater frequency of the IL-4 receptor alpha chain (IL-4RA) ile75val polymorphism, which was associated with greater CD23 expression by both CD19^+^ and CD19^+^CD86^+^ B cells stimulated with IL-4 (Knutsen et al., 2010). As well, the moderate-to-severe group showed a reduced allele frequency of HLA-DQB1*03, suggesting its potential role in asthma severity where fungal sensitization is involved (Knutsen et al., 2010). Altogether, these studies highlight the multifactorial association between fungi and asthma, with environmental fungal exposures, innate and adaptive immune mechanisms, genetic susceptibilities, and microbiome features at play.

## Fungi as Targets for Asthma Treatment and Control

Given the important role of fungi in mediating allergic airway disease, it is imperative to consider them as potential components in strategies to improve symptoms and disease severity. Studies have shown that antibiotic treatment in mice, resulting in a dysbiotic bacterial and fungal gut microbiome, increased susceptibility to fungi driven airway disease (Noverr et al., 2004; Noverr et al., 2005; Kim et al., 2014; Skalski et al., 2018). These findings suggest that the amelioration of fungal dysbiosis may be valuable in the prevention or treatment of fungi-associated asthma.

Due to the severity of fungi-associated asthma and its unique underlying immunopathology, specialized therapeutics must be developed aimed at targeting fungal colonizers and improve disease status and management. While the use of broad-spectrum antifungals may seem helpful, some studies have described deleterious effects to the mycobiome, resulting in host immune dysregulation. Treatment of mice with fluconazole resulted in fungal dysbiosis in the gut and enhanced allergic airway responses when mice were challenged in an HDM model of airway inflammation (Wheeler et al., 2016). Fluconazole selectively depleted *Candida* spp. and allowed for the expansion of *Aspergillus*, *Wallemia*, and *Epicoccum* taxa. A similar induction of fungal dysbiosis was seen with amphotericin-B treatment (Wheeler et al., 2016). Further, these three expanded fungal taxa were verified as causative agents for enhanced airway responses to HDM, as mice that received supplementation with these taxa displayed similar results to those treated with antifungal drugs (Wheeler et al., 2016). Results from our lab also demonstrated that a one-week-long treatment with fluconazole to mouse dams during the pre-weaning period was sufficient to persistently alter the gut fungal ecosystem of mouse pups colonized with defined communities of yeasts and bacteria (van Tilburg Bernardes et al., 2020). Similarly to early life antibiotic treatment, antifungal-induced microbiome perturbations led to broad systemic immune alterations and increased susceptibility to OVA-induced airway inflammation, characterized by elevated BAL eosinophilia (van Tilburg Bernardes et al., 2020). These results strongly suggest that fungal-derived signals are important during immune development, and that the effects of antifungals are akin to those of antibiotics on the bacterial microbiome, inducing widespread microbiome perturbations that can trigger immune dysregulation and increase susceptibility to asthma.

Nonetheless, targeting of specific fungal colonizers may be a therapeutic avenue to be explored for fungal asthma or SAFS. A randomized clinical trial to investigate the use of oral antifungal, itraconazole, in patients with SAFS was first carried out in 2009 by Denning et al. (2009). In this trial, 60% of patients reported an improved quality of life relative to the placebo group, indicating that this may be a viable therapeutic option for patients suffering from fungal asthma (Denning et al., 2009). Though, it remained unclear whether this effect was due to the depletion of fungi or immunological effect of itraconazole in dampening of Th2 responses. Despite these findings, the use of antifungal drugs for treatment of fungal asthma has not been widely accepted due to the difficulty in identifying the specific subgroup of SAFS patients that would truly benefit from the treatment (Parulekar et al., 2015). The European Respiratory Society/American Thoracic Society Task Force only recommends antifungal therapy for allergic bronchopulmonary aspergillosis, and advises against the treatment for SAFS due to limited evidence of treatment efficacy within this group (Chung et al., 2014). Research into the use of antifungal therapy for fungal-associated asthma is ongoing and a recent retrospective cohort study of 41 patients with culture-proven airway mycosis by Li E. et al. (2018) reported improved asthma control, reduced eosinophilia, and lowered serum IgE associated with antifungal treatment. Larger scale clinical trials are needed to truly establish the benefit and underlying mechanisms by which beneficial results are conferred, along with the safety of antifungals in patients suffering from fungi-associated asthma, as viable treatment options are greatly needed.

An alternative approach to develop fungi-associated asthma therapeutics is to target specific immune responses elicited by fungi in order to improve symptoms and disease severity. One example is inhibitors of fungal proteases, which have been shown to reduce the airway damage associated with fungal proteases (Balenga et al., 2015). Additionally, the improvement in Th1 and Th17 responses following IL-1RA treatment seen by Godwin et al. (2019) suggests that drugs such as Anakinra (recombinant human IL-1RA), which has been recommended for treatment of other inflammatory diseases, including rheumatoid arthritis, may be a treatment option in patients suffering from fungi-associated asthma. A phase I clinical study found that Anakinra significantly reduced inhaled endotoxin-induced airway neutrophilia, along with IL-1β, IL-6, and IL-8, in healthy volunteers relative to the control (Hernandez et al., 2015). As neutrophils have been linked to fungi-associated asthma severity (Park et al., 2006), the findings from this phase I trial are a promising area for future research. Furthermore, the same investigators from this phase I trial (Park et al., 2006) also have two ongoing randomized clinical trials exploring the efficacy of Anakinra as a therapeutic option for allergic manifestation in asthmatic patients (NCT03513471 and NCT03513458). These ongoing trials have great potential to uncover Anakinra’s ability to safely control early and/or late phases of asthma exacerbation episodes. Specific targeting of these immunological mechanisms, among others, may be a viable therapeutic option though more research must be carried out to determine safety and viability in treatment of asthmatic patients.

## Conclusions and Future Directions

Fungi, viruses, archaea, and bacteria coinhabit all terrestrial and aquatic environmental niches, including the mammalian mucosae, engaging in diverse ecological interkingdom relationships that shape the overall composition of microbiomes, and their relationship with the host (Frey-Klett et al., 2011; Sharma et al., 2019; van Tilburg Bernardes et al., 2020). Non-bacterial members of the microbiome, are increasingly being recognized as important players in asthma development and manifestation (Barcik et al., 2020), highlighting the importance of multi-kingdom microbial interactions in the pathophysiology of this disease. Nevertheless, the characterization of the direct implications of fungi, and other members of the microbiome in disease manifestation is still nascent. Microbiome studies should move beyond surveying only bacteria, and consider the multi-kingdom nature of these important ecosystems.

Despite continuous advancements in sequencing technologies, there are still important challenges to attain a sensitive and precise assessment of the mycobiome within complex communities (Tiew et al., 2020). Improvements remain necessary in fungal taxonomic databases and in bioinformatic algorithms that can efficiently account for the higher variability in read length and higher rate of genetic insertions and deletions in the regions used in most mycobiome sequencing approaches (i.e., ITS1 and ITS2 regions). Further, there is very little knowledge on the functional role of fungi in the microbiome, how this impacts overall metabolic output, and the community’s response to perturbation (e.g., antimicrobials), which are crucial to further understand the role of the microbiome in asthma.

While strong evidence suggests that both the lung and gut mycobiome are important players in asthma, the specific fungal-driven cellular and molecular mechanisms of disease are poorly understood. Despite this, our current state of knowledge renders great potential for microbiome-derived approaches aimed at preventing and treating this disease. To this aim, research must seek to further elucidate the role of fungi in early immune education events and characterize the specific cellular and molecular mechanisms by which early-life fungal dysbiosis promotes or protects from immune responses conducive of asthma. This is especially evident in lung mycobiome studies, as no prospective study has investigated early life airway alterations that may be associated with asthma development in children, further limiting mechanistic studies aimed at determining the causal role of airway colonizing fungi in disease pathogenesis.

Evidence to date is sufficient to disprove the assumption that all fungal exposures, whether from the environment or the microbiome, are detrimental to health (Tischer et al., 2011; Behbod et al., 2015). Defining which species of fungi are protective or harmful for asthma development and/or exacerbations is an attainable goal if more prospective longitudinal studies survey fungal organisms within the microbiome and the environment of the human population studied.

Moreover, it will be crucial to consider that environmental exposures to allergenic moulds (e.g., *Alternaria*, *Aspergillus*, and *Penicillium*) may become even more prevalent in the face of ongoing climate change. Moulds thrive in moist and warm environments (Weinhold, 2007), thus worldwide rising temperatures may favor the growth of known allergenic fungi, and the emergence of new ones. A recent meta-analysis of 341 publications highlighted how soil microbial communities are sensitive to global change factors (Zhou et al., 2020). Based on a mixed-effect model, the investigators reported that diversity of fungal communities are impacted by changing environmental conditions (Zhou et al., 2020). Hence, aspects of climate change have the potential to alter the dynamics of environmental fungi, which may in turn impact development and/or exacerbation rates of fungi-associated asthma, including changes in precipitation and nutrient availability (Zhou et al., 2020), atmospheric CO_2_ levels (Klironomos et al., 1997), and shifts in the duration of seasons (Cecchi et al., 2010), overall contributing to increased levels of fungal aeroallergens.

Finally, we need a more comprehensive understanding on how antimicrobial drugs and other early-life microbiome alterations (e.g., Caesarean sections and formula feeding) impact the infant mycobiome, and which of these alterations may be conducive of asthma. Including fungi in these studies will provide a more ecologically sound framework to design effective probiotic consortia capable of remediating the microbiome perturbations and host immune dysregulation that continue to increase asthma risk around the world.

## Author Contributions

EvTB, MWG, and M-CA formulated the concept for this review. EvTB and MWG wrote the first draft. All authors contributed to the article and approved the submitted version.

## Funding

EvTB is funded by the Eyes High Doctoral Recruitment Scholarship. MWG is funded by the Alberta Children's Hospital Research Institute Graduate Scholarship. M-CA is funded by the Cumming School of Medicine, the Alberta Children Hospital Research Institute, the Snyder Institute of Chronic Diseases, the Canadian Institutes for Health Research, the Sick Kids Foundation, the W. Garfield Weston Foundation, The Koopmans Research Fund, and the Canadian Lung Association.

## Conflict of Interest

The authors declare that the research was conducted in the absence of any commercial or financial relationships that could be construed as a potential conflict of interest.
